# Transcriptome assembly and quantification from Ion Torrent RNA-Seq data

**DOI:** 10.1186/1471-2164-15-S5-S7

**Published:** 2014-07-14

**Authors:** Serghei Mangul, Adrian Caciula, Sahar Al Seesi, Dumitru Brinza, Ion Mӑndoiu, Alex Zelikovsky

**Affiliations:** 1 Department of Computer Science, University of California, Los Angeles, CA, USA; 2 Department of Computer Science, Georgia State University, Atlanta, GA, USA; 3 Deptment of Computer Science & Engineering, University of Connecticut, Storrs, CT, USA; 4 Ion Bioinformatics, Life Technologies Corporation, Foster City, CA, USA

## Abstract

**Background:**

High throughput RNA sequencing (RNA-Seq) can generate whole transcriptome information at the single transcript level providing a powerful tool with multiple interrelated applications including transcriptome reconstruction and quantification. The sequences of novel transcripts can be reconstructed from deep RNA-Seq data, but this is computationally challenging due to sequencing errors, uneven coverage of expressed transcripts, and the need to distinguish between highly similar transcripts produced by alternative splicing. Another challenge in transcriptomic analysis comes from the ambiguities in mapping reads to transcripts.

**Results:**

We present MaLTA, a method for simultaneous transcriptome assembly and quantification from Ion Torrent RNA-Seq data. Our approach explores transcriptome structure and incorporates a maximum likelihood model into the assembly and quantification procedure. A new version of the IsoEM algorithm suitable for Ion Torrent RNA-Seq reads is used to accurately estimate transcript expression levels. The MaLTA-IsoEM tool is publicly available at: http://alan.cs.gsu.edu/NGS/?q=malta

**Conclusions:**

Experimental results on both synthetic and real datasets show that Ion Torrent RNA-Seq data can be successfully used for transcriptome analyses. Experimental results suggest increased transcriptome assembly and quantification accuracy of MaLTA-IsoEM solution compared to existing state-of-the-art approaches.

## Background

Massively parallel whole transcriptome sequencing, commonly referred to as RNA-Seq, and its ability to generate full transcriptome data at the single transcript level, provides a powerful tool with multiple interrelated applications, including transcriptome assembly [1-4], gene and transcript expression level estimation [5-8], also known as transcriptome quantification, studying trans- and cis-regulatory effects [9], studying parent-of-origin effects [9-11], and calling expressed variants [12].

RNA-Seq has become the technology of choice for performing transcriptome analysis, rapidly replacing array-based technologies [13]. The Ion Torrent technology offers the fastest sequencing protocol for RNA-Seq experiments able to sequence whole transcriptome in few hours [14]. Most current research using RNA-Seq employs methods that depend on existing transcriptome annotations. Unfortunately, as shown by recent targeted RNA-Seq studies [15], existing transcript libraries still miss large numbers of transcripts. The incompleteness of annotation libraries poses a serious limitation to using this powerful technology since accurate normalization of RNA-Seq data critically requires knowledge of expressed transcript sequences [5-8]. Another challenge in transcriptomic analysis comes from the ambiguities in read/tag mapping to transcripts. Ubiquitous regulatory mechanisms such as the use of alternative transcription start and polyadenylation sites, alternative splicing, and RNA editing result in multiple messenger RNA (mRNA) isoforms being generated from a single genomic locus. Most prevalently, alternative splicing is estimated to take place for over 90% of the multi-exon human genes across diverse cell types [8], with as much as 68% of multi-exon genes expressing multiple isoforms in a clonal cell line of colorectal cancer origin [16]. The ability to reconstruct full length transcript sequences and accurately estimate their expression levels is widely believed to be critical for unraveling gene functions and transcription regulation mechanisms [17].

Here, we focus on two main problems in transcriptome analysis, namely, transcriptome assembly and quantification. Transcriptome assembly, also known as novel transcript discovery or reconstruction, is the problem of assembling the full length transcript sequences from the RNA sequencing data. Assembly can be done *de novo *or it can be assisted by existing genome and transcriptome annotations. Transcriptome quantification is the problem of estimating the expression level of each transcript. In the remainder of this section we give a brief description of the common protocols used for mRNA sequencing.

### RNA-Seq protocol

**Figure 1:**
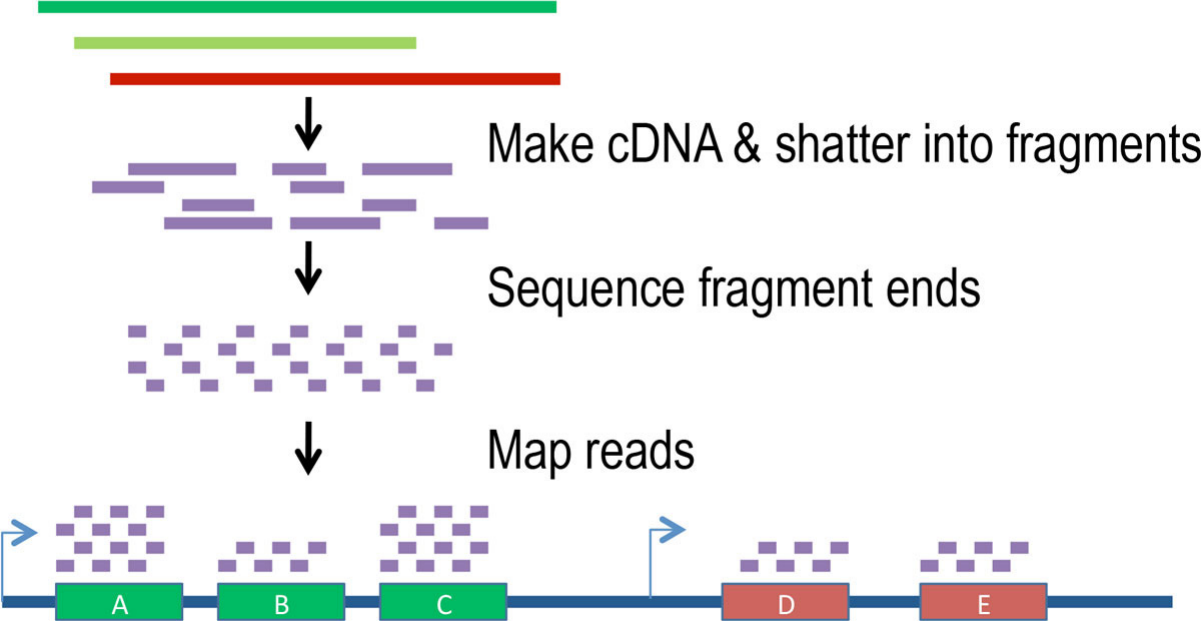
**A schematic representation of the RNA sequencing protocol**.

RNA-Seq uses next generation sequencing technologies, such as SOLiD [18], 454 [19], Illumina [20], or Ion Torrent [21]. Figure 1 depicts the main steps in an RNA-Seq experiment, ending with the first step of analysis which is typically mapping the data to a reference. The mRNA extracted from a sample is converted to cDNA using reverse transcription and sheared into fragments. Fragments with lengths within a certain range are selected, and ligated with sequencing adapters. This is usually followed by an amplification step after which one or both ends of the cDNA fragments are sequenced to produce either single or paired-end reads. cDNA synthesis and adapter ligation can be done in a strand-specific manner, in which case the strand of each read is known; this is commonly referred to as directional sequencing. In the more common non-directional RNA-Seq protocols strand specificity is not maintained. The specifics of the sequencing protocols vary from one technology to the other. In particular, the length of produced reads varies depending on the technology, with newer high-throughput technologies typically producing longer reads.

### Related work

Transcriptome assembly and quantification from RNA-Seq data has been the focus of much research in recent years. The sequences of novel transcripts together with their expression levels can be inferred from deep RNA-Seq data, but this is computationally challenging due to the short length of the reads, high percentage of sequencing errors, uneven coverage of expressed transcripts, and the need to distinguish between highly similar transcripts produced by alternative splicing. A number of methods address the problem of transcriptome assembly and quantification from RNA sequencing data. Methods for transcriptome assembly fall into three categories: "genome-guided", "genome-independent" and "annotation-guided" methods [22]. Genome-independent methods such as Trinity [23] or transAbyss [24] directly assemble reads into transcripts. A commonly used approach for such methods is the de Brujin graph [25] utilizing "k-mers". The use of genome-independent methods becomes essential when there is no trusted genome reference that can be used to guide assembly. On the other end of the spectrum, annotation guided methods [26-28] make use of available information in existing transcript annotations to aid in the discovery of novel transcripts. RNA-Seq reads can be mapped onto the reference genome, reference annotations, exon-exon junction libraries, or combinations thereof, and the resulting alignments are used to assemble transcripts.

Many transcriptome reconstruction methods fall in the genome-guided category. They typically start by mapping sequencing reads onto the reference genome, using spliced alignment tools, such as TopHat [29] or SpliceMap [30]. The spliced alignments are used to identify putative exons, splice junctions and transcripts that explain the alignments. While some methods aim to achieve the highest sensitivity, others work to predict the smallest set of transcripts explaining the given input reads. Furthermore, some methods aim to reconstruct the set of transcripts that would insure the highest quantification accuracy. Scripture [1] construct a splice graph from the mapped reads and reconstructs transcripts corresponding to all possible paths in this graph. It then uses paired-end information to filter out some transcripts. Although Scripture achieves very high sensitivity, it may predict a lot of incorrect isoforms. The method of Trapnell et al. [4,31], referred to as Cufflinks, constructs a read overlap graph and reconstructs transcripts using a minimal size path cover via a reduction to maximum matching in a weighted bipartite graph. TRIP [3] uses an integer programming model where the objective is to select the smallest set of putative transcripts that yields a good statistical fit between the fragment length distribution empirically determined during library preparation and fragment lengths implied by mapping read pairs to selected transcripts. IsoLasso [32] uses the LASSO [33] algorithm, and it aims to achieve a balance between quantification accuracy and predicting the minimum number of transcripts. It formulates the problem as a quadratic program, with additional constraints to ensure that all exons and junctions supported by the reads are included in the predicted isoforms. CLIIQ [34] uses an integer linear programming solution that minimizes the number of predicted isoforms explaining the RNA-Seq reads while minimizing the difference between estimated and observed expression levels of exons and junctions within the predicted isoforms. Traph [35] proposed a method based on network flows for a multiassembly problem arising from transcript identification and quantification with RNA-Seq. Another method, CLASS [36] uses local read coverage patterns of RNA-seq reads and contiguity constraints from read pairs and spliced reads to predict transcripts from RNA-Seq data. iReckon [37] is a method for simultaneous determination of the transcripts and estimation of their abundances. This probabilistic approach incorporates multiple biological and technical phenomena, including novel isoforms, intron retention, unspliced pre-mRNA, PCR amplification biases, and multi-mapped reads. iReckon utilizes regularized Expectation-Maximization to accurately estimate the abundances of known and novel transcripts.

## Methods

### Spliced alignment

Alignment of RNA-Seq reads onto the reference genome, reference annotations, exon-exon junction libraries, or combinations thereof is the first step of RNA-Seq analyses, unless none of these are available in which case it is recommended to use *de novo *assembly methods [23,24]. The best mapping strategy depends on the purpose of RNA-Seq analysis. If the focus of the study is to estimate transcripts and gene expression levels rather then discover new transcripts then it is recommended to map reads directly onto the set of annotated transcripts using a fast tool for ungapped read alignment. To be able to discover new transcriptional variants one should map the reads onto the reference genome. Recently, many bioinformatics tools, called spliced read aligners, have been developed to map RNA-Seq reads onto a reference genome [29,30]. Alternatively, RNA-Seq reads can be mapped onto the genome using a local alignment tool such as the Ion Torrent mapper, TMAP. Both spliced alignments and local alignments can be used to detect novel transcriptional and splicing events including exon boundaries, exon-exon junctions, gene boundaries, transcriptional start (TSS) and transcription end sites (TES).

In our experiments we used TopHat [29] with default parameters. For assessing transcriptome quantification accuracy Ion Torrent reads from cancer datasets were mapped on the External RNA Controls Consortium (ERCC) RNA spike-in controls reference [38] with added polyA tails of 200 bp using TMAP. Reads for the MAQC datasets were mapped onto Ensembl known transcripts with added polyA tails of 200 bp, also using TMAP.

### Splice graph and putative transcripts

**Figure 2:**
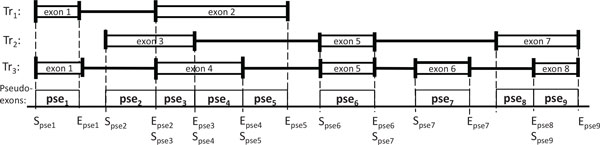
**Pseudo-exons**. An example of three transcripts, ***Tr*_1_**, ***Tr*_2 _**and ***Tr*_3_**. Each transcript is represented as a set of exons. Pseudo-exons are regions of a gene between consecutive transcriptional or splicing events. ***S_psej _***and ***E_psej _***represent the starting and ending position of pseudo-exon ***j***, respectively.

Typically, a gene can express multiple mRNA transcripts due to alternative transcriptional or splicing events including alternative first exon, alternative last exon, exon skipping, intron retention, alternative 5' splice site (A5SS), and alternative 3' splice site (A3SS) [39]. To represent such alternative transcripts, a gene is processed as a set of so called 'pseudo-exons' based on alternative variants obtained from aligned RNA-Seq reads. A pseudo-exon is a region of a gene between consecutive transcriptional or splicing events, i.e., starting or ending of an exon, as shown in Figure 2. Hence, every gene consists of a set of non-overlapping pseudo-exons. This gene representation lets us easily enumerate all possible transcripts of a gene. To generate the set of putative transcripts, we first create a splice graph based on pseudo-exon boundaries and splice junctions.

**Figure 3:**
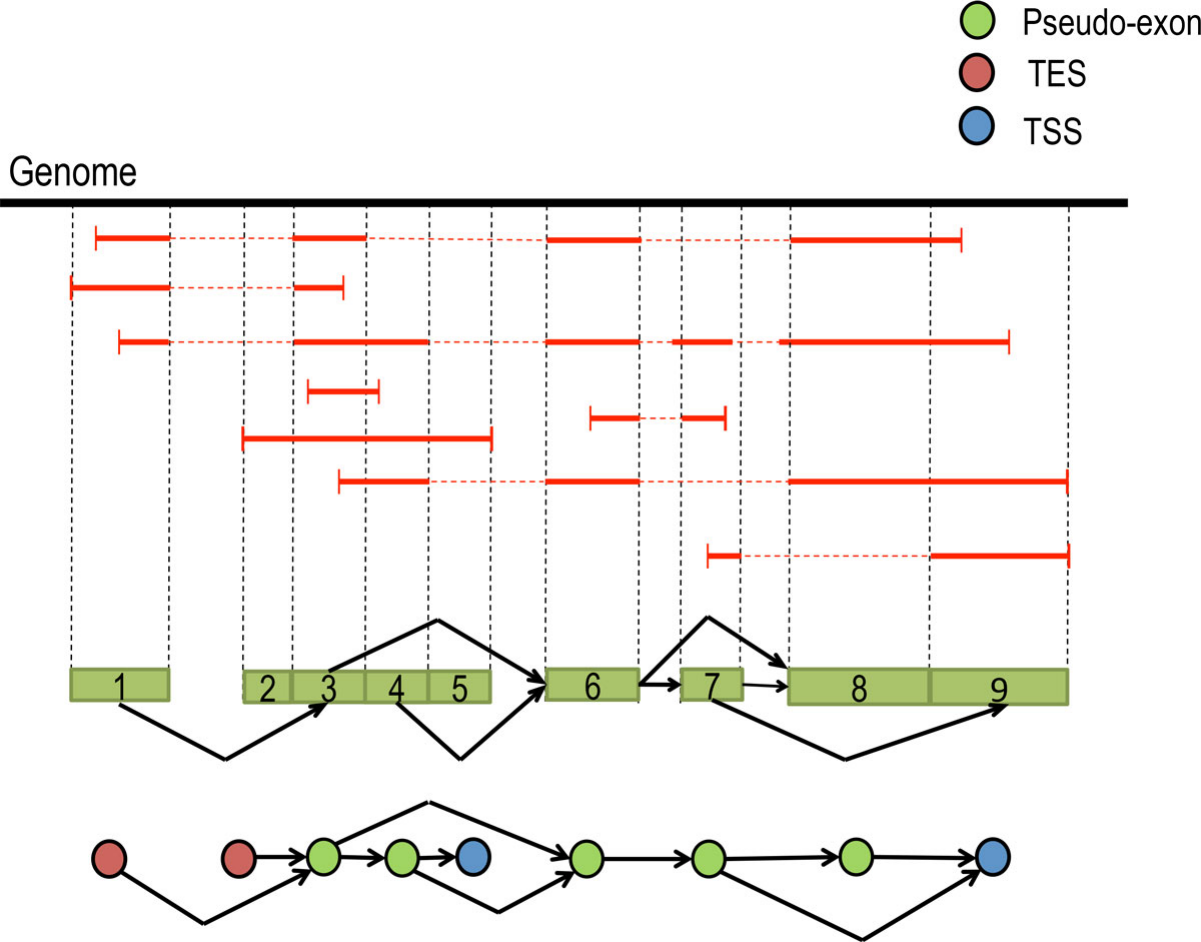
**Splice graph**. The red horizontal lines represent single reads. Reads interrupted by dashed lines are spliced reads. Each vertex of the splice graph corresponds to a pseudo-exon and each directed edge corresponds to a (splice) junction between two pseudo-exons. Red vertices of the slice graph serve as transcription start sites (TSS). Blue vertices - transcription end sites (TES).

The splice graph is a directed acyclic graph (Figure 3) whose vertices represent pseudo-exons and edges represent pairs of pseudo-exons immediately following one another in at least one transcript (which is witnessed by at least one spliced read). Both splice junctions and pseudo-exon boundaries are inferred from read alignments. To construct the splice graph, MaLTA infers splice junctions from gapped alignments of RNA-seq reads. Next, inferred splice junctions are used to partition the reference genome into a set of non-overlapping segments, which are classified as (a) intron, (b) pseudo-exon, or (c) combination of both. It is easy to classify a segment as pseudo-exon if it is entirely covered, and as intron in case it is entirely uncovered. In case of partial coverage we require 80% of the segment to be covered to be classified as pseudo-exon, otherwise it is classified as (a) or (c). Segments containing a combination of introns and exons most likely contain gene boundaries. In this case we identify islands of coverage inside the segment. A segment may contain several coverage islands which correspond to single exon genes.

After constructing the splice graph, MaLTA enumerates all maximal paths using a depth-first-search algorithm. These paths correspond to putative transcripts. Note that a gene with *n *pseudo-exons may have as many as 2*^n ^*− 1 possible candidate transcripts, each composed of a subset of the *n *pseudo-exons. The next subsection presents a maximum likehood transcriptome assembly and quantification algorithm that selects a minimal subset of candidate transcripts that best fits the observed RNA-Seq reads. The key ingredient is an expectation-maximization algorithm for estimating expression levels of candidate transcripts.

### Maximum likehood transcriptome assembly

Existing transcriptome assembly methods [3,4] use read pairing information and fragment length distribution to accurately assemble the set of transcripts expressed in a sample. This information is not available for current Ion Torrent technology, which can make it challenging to assemble transcripts. The Ion Torrent PGM platform is able to produce single reads with read length in 50-300 bp range. Our approach is to simultaneously explore the transcriptome structure and perform transcriptome quantification using a maximum likelihood model. MaLTA starts from the set of putative transcripts and selects the subset of this transcripts with the highest support from the RNA-Seq data. Maximum likelihood estimates of putative transcripts are computed using an Expectation Maximization (EM) algorithm which takes into account alternative splicing and read mapping ambiguities. EM algorithms are currently the state-of-the-art approach to transcriptome quantification from RNA-Seq read, and have been proven to outperform count-based approaches. Several independent implementations of EM algorithm exist in the literature [7,40].

We developed a new version of IsoEM [7] suitable for Ion Torrent RNA-Seq reads. IsoEM is an expectation-maximization algorithm for transcript frequency estimation. It overcomes the problem of reads mapping to multiple transcripts using iterative framework which simultaneously estimates transcript frequencies and imputes the missing origin of the reads. A key feature of IsoEM, is that it exploits information provided by the distribution of insert sizes, which is tightly controlled during sequencing library preparation under current RNA-Seq protocols. In [7], we showed that modeling insert sizes is highly beneficial for transcript expression level estimation even for RNA-Seq data consisting of single reads, as in the case of Ion Torrent. Modeling insert sizes contributes to increased estimation accuracy by disambiguating the transcript of origin for the reads. In IsoEM, insert lengths are combined with base quality scores, and, if available, strand information to probabilistically allocate reads to transcripts during the expectation step of the algorithm. Since most Ion Torrent sequencing errors are insertions and deletions, we developed a version of IsoEM capable of handling insertions and deletions in read alignments.

The main idea of the MaLTA approach is to cover all trancriptional and splicing variants presented in the sample with the minimum set of putative transcripts. We use the new version of the IsoEM algorithm described above to estimate expression levels of putative transcripts. Since IsoEM is run with all possible candidate transcripts, the number of transcripts that are predicted to have non-zero frequency can still be very large. Instead of selecting all transcripts with non zero frequency, we would like to select a small set of transcripts that explain all observed splicing events and have highest support from the sequencing data. To realize this idea we use a greedy algorithm which traverses the list of candidate transcripts sorted in descending order by expression level, and selects a candidate transcript only if it contains a transcriptional or splicing event not explained by the previously selected transcripts.

## Results and discussions

We evaluated the accuracy of the MaLTA-IsoEM approach on both simulated and real human RNA-Seq data. The human genome sequence (hg18, *NCBI *build 36) was downloaded from *UCSC *together with the KnownGenes transcripts annotation table. Genes were defined as clusters of known transcripts defined by the GNFAtlas2 table. In our simulation experiments, we simulate reads together with spliced alignments to the genome; these alignments are provided to all compared methods. We varied the length of single-end reads, which were randomly generated per gene by sampling fragments from known transcripts. All the methods were compared on datasets with various read length, i.e., 50 bp, 100 bp, 200 bp, and 400 bp. Expression levels of transcripts inside each gene cluster followed uniform and geometric distributions. To address library preparation process of RNA-Seq experiment we simulated fragment lengths from a normal probability distribution with different means and 10% standard deviation.

All reconstructed transcripts were matched against annotated transcripts. As in [4] and [32], two transcripts were assumed to match if and only if internal exon boundaries coordinates (i.e. all exons coordinates except the beginning of the first exon and the end of the last exon) were identical. We use sensitivity and positive predictive value (PPV) to evaluate the performance of different assembly methods. Sensitivity is defined as the proportion of assembled transcripts that match annotated transcripts, i.e., *sensitivity *= *TP/*(*TP *+ *FN*). Positive predictive value (PPV) is defined as the proportion of annotated transcript sequences among assembled sequences, i.e., *PPV *= *TP/*(*TP *+ *FP*).

Transcriptome quantification accuracy was evaluated by comparing RNA-Seq estimates with TaqMan qRT-PCR measurements [41] or External RNA Controls Consortium (ERCC) RNA spike-in controls [38]. The coefficient of determination (*R*^2^) between the (qRT-PCR/ERCC) and Fragment Per Kilobase of exon length per Million reads (FPKM) estimates was used as accuracy measure.

### Comparison on simulated RNA-Seq data

In this section, we use sensitivity and PPV defined above to compare the MaLTA to other transcriptome assembly tools. The most recent versions of Cufflinks (version 2.1.1) [4] and IsoLasso (v 2.6.0) [2] with the default parameters are used for performance comparison. We explore the influence of read and fragment length on performance of assembly methods.

Table 1 reports sensitivity and PPV of transcriptome assembly for reads of length 400 bp, simulated assuming both uniform and geometric expression of transcripts. MaLTA significantly outperforms the other methods, achieving both sensitivity and PPV of over 75% for all datasets. For all methods the difference in accuracy between datasets generated assuming uniform and geometric distribution is small, with the latter one typically having a slightly worse accuracy. Thus, in the interest of space we present remaining results for datasets generated using uniform distribution.

**Table 1:** Sensitivity and PPV comparison between methods on datasets simulated assuming uniform, respectively geometric expression of transcripts, with reads length 400 bp, mean fragment length 450 bp and 10% standard deviation.

Transcript expression	Methods	# assembled transcripts	# confirmed annotated transcripts	Sensitivity (%)	PPV (%)
Uniform	Cufflinks	18,582	12,909	51.06	69.47
	MaLTA	23,706	18,698	76.69	78.87
	IsoLasso	21,441	15,693	63.52	73.19

Geometric	Cufflinks	17,377	12,449	50.21	71.64
	MaLTA	22,931	18,293	76.05	79.77
	IsoLasso	20,816	15,308	62.83	73.54

There is a strong correlation between the number of splicing events within the gene and the number of annotated transcripts. A high number of splicing events leads to increased number of candidate transcripts, which makes the selection process more difficult. To explore the behavior of the methods depending on number of transcripts per gene we divided all genes into categories according to the number of annotated transcripts and calculated the sensitivity and PPV within each such category.

**Figure 4:**
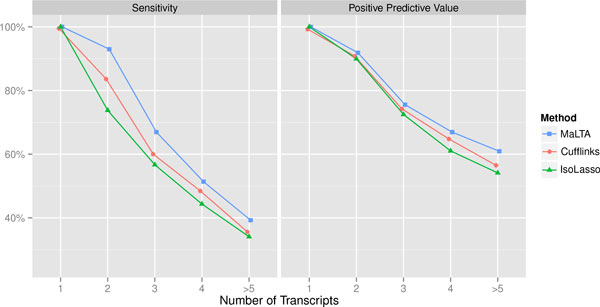
**Sensitivity and PPV comparison between methods for groups of genes with n transcripts on simulated datasets with mean fragment length 250 bp, 10% standard deviation, and read length of 100 bp**.

Figures 4(a)-4(b) compare the performance of three methods (Cufflinks, IsoLasso, MaLTA) on simulated data with respect to the number of transcripts per gene. Note that sensitivity and PPV (Figure 4) for single-transcript genes is 100% for all methods and is excluded from consideration. MaLTA achieves equivalent or better results in both sensitivity and PPV for all categories.

Table 2 compares assembly accuracy of Cufflinks, IsoLasso, and MaLTA for different combinations of read and fragment lengths: (50 bp,250 bp), (100 bp,250 bp), (100 bp,500 bp), (200 bp,250 bp), (400 bp,450 bp). The results show that MaLTA provide 5-15% improvement in sensitivity and 1-10% improvement in PPV.

**Table 2:** Sensitivity and PPV comparison between methods for different combinations of read and fragment lengths: (50 bp, 250 bp), (100 bp, 250 bp), (100 bp, 500 bp), (200 bp, 250 bp), (400 bp, 450 bp).

Read	Fragment length	Methods	# assembled transcripts	# confirmed annotated transcripts	Sensitivity (%)	PPV (%)
50	250	Cufflinks	18,483	14,179	67.36	76.71
		MaLTA	20,036	15,894	75.53	79.33
		IsoLasso	19,422	15,287	70.66	78.71

100	250	Cufflinks	17,981	14,073	69.30	78.27
		MaLTA	19,405	15,539	76.72	80.08
		IsoLasso	16,864	12,802	62.60	75.91
	500	Cufflinks	18,958	14,757	67.19	77.84
		MaLTA	20,481	16,326	74.73	79.71
		IsoLasso	17,979	13,428	60.29	74.69

200	250	Cufflinks	20,435	15,637	66.57	76.52
		MaLTA	21,823	17,265	74.89	79.11
		IsoLasso	19,422	15,287	70.66	78.71

400	450	Cufflinks	18,483	14,179	67.36	76.71
		MaLTA	20,036	15,894	75.53	79.33
		IsoLasso	19,422	15,287	70.66	78.71

### Comparison on Ion Torrent cancer and MAQC RNA-Seq datasets

For this study, we compared MaLTA and Cufflinks on 3 cancer datasets downloaded from the Ion Community website: GOG-382 (HepG2 - hepatocellular carcinoma), DID-416 (K562 - myelogenous leukemia) and DID-413 (MCF-7 - breast ductal carcinoma). Comparison with IsoLasso on the real datasets is omitted due to technical problems (IsoLasso results were consistently incomparable to other methods). Reads were mapped to the hg18 reference genome using TopHat2 (with default parameters) which is able to produce spliced alignment used by transcriptome assembly tools (Table 3).

**Table 3:** Read mapping statistics and read length for Ion Torrent HeLa datasets.

Dataset	Type of cancer	# reads	# mapped reads	Mean read length (bp)
GOG-382	hepatocellular carcinoma	4,964,525	1,284,796	94

DID-416	myelogenous leukemia	5,024,097	1,115,392	89

DID-413	breast ductal carcinoma	3,134,849	690,870	108

Reads are mapped to hg18 reference genome using TopHat2 with default parameters.

**Figure 5:**
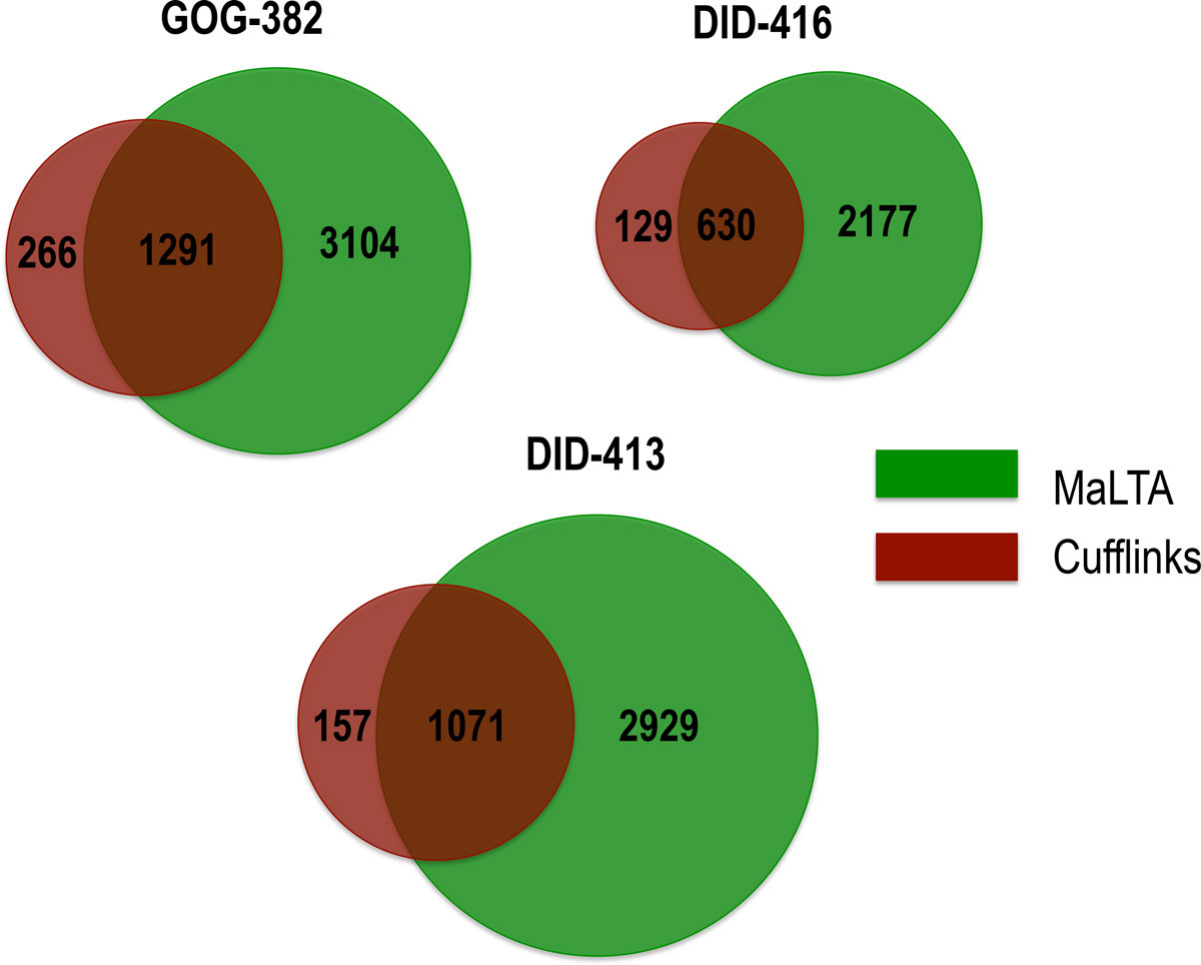
**Consistency of transcriptome assembly**. Number of identified annotated transcripts confirmed by both methods for GOG-382,DID-416 and DID-413 datasets.

Although UCSC annotations are known to be incomplete, we expect a significant proportion of assembled transcripts to be consistent with these annotations. Thus, the performance of transcriptome assembly methods was evaluated by the total number of assembled transcripts matching UCSC annotations. Table 4 gives the results obtained by MaLTA and Cufflinks on DID-413, DID-416 and GOG-382 datasets. Both methods assemble highest number of transcripts confirmed by reference annotations for GOG-382 dataset. Cufflinks and MaLTA respectively were able to assemble 13,887 and 16,143 transcripts, of which 1,557 and 4,395 are known annotated transcripts. A large number of identified annotated transcripts were confirmed by both methods (Figure 5). The GOG-382 dataset contains the highest number of annotated transcripts confirmed by both methods; among identified annotated transcripts 1,291 transcripts were confirmed by both methods.

**Table 4:** Performance comparison of transcriptome assembly between Cufflinks and MaLTA for Ion Torrent HeLa datasets.

	DID-413		DID-416		GOG-382	
	**# assembled transcripts**	**# confirmed annotated transcripts**	**# assembled transcripts**	**# confirmed annotated transcripts**	**# assembled transcripts**	**# confirmed annotated transcripts**

MaLTA	15,109	4,000	9,908	2,807	16,143	4,395
Cufflinks	12,100	1,228	7,419	759	13,887	1,557

Assembled transcripts are matched against UCSC hg18 reference annotations.

**Figure 6:**
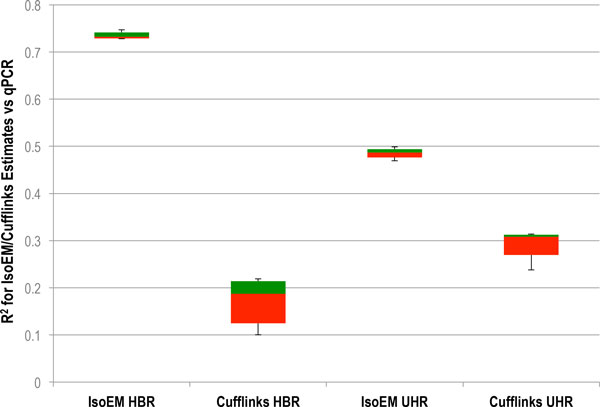
**Correlation of estimates obtained by both IsoEM and Cufflinks with qPCR measurments for HBRR and UHRR datasets**. The red color represents the 2nd quartile and the green color represents the 3rd quartile.

To evaluate transcriptome quantification accuracy of the methods we ran IsoEM and Cufflinks on Ion Torrent RNA-Seq data generated from two commercially available reference RNA samples that have been well-characterized by quantitative real time PCR (qRT-PCR) as part of the MicroArray Quality Control Consortium (MAQC); namely the Ambion Human Brain Reference RNA, Catalog #6050), henceforth referred to as HBRR and the Stratagene Universal Human Reference RNA (Catalog #740000), henceforth referred to as UHRR. We used five HBRR datasets and five UHRR datasets for the comparison. To assess accuracy, gene expression estimates obtained from RNA-Seq data were compared against those obtained from TaqMan qRT-PCR measurements (GEO accession GPL4097) collected as part of the MAQC project. As described in [41], each TaqMan Assay was run in four replicates for each measured gene. POLR2A (ENSEMBL id ENSG00000181222) was chosen as the reference gene and each replicate CT was subtracted from the average POLR2A CT to give the log2 difference (delta CT). For delta CT calculations, a CT value of 35 was used for any replicate that had CT *>*35. The normalized expression value for gene *g *was computed as 2(CT of POLR2A)-(CT of *g*), and the average of the qPCR expression values in the four replicates was used as the ground truth. Mapping gene names to Ensembl gene IDs using the HUGO Gene Nomenclature Committee (HGNC) database resulted in TaqMan qPCR expression levels for 832 Ensembl genes. Tables 5 and 6 show statistics for the size, number of mapped reads, and accuracy of gene expression levels estimated by IsoEM for each of the 10 datasets as well as the combined reads for each sample. Figure 6 presents a comparison between IsoEM and Cufflinks results. IsoEM estimates correlate better with qPCR measurements compared to Cufflinks. Additionally, IsoEM estimates have less variability across different Ion Torrent runs.

**Table 5:** Read mapping statistics and correlation between gene expression levels estimated by IsoEM and qPCR measurement for Ion Torrent UHRR dataset.

Run	# reads	# mapped reads	*R* ^2^
POZ-125 268	1,601,962	1,103,357	0.489
DID-144 283	1,990,213	1,368,073	0.487
POZ-126 269	1,800,034	1,291,935	0.469
GOG-140 284	2,052,587	1,452,006	0.499
POZ-127 270	2,263,519	1,615,623	0.484

All runs	9,708,315	6,830,990	0.485

**Table 6:** Read mapping statistics and correlation between gene expression levels estimated by IsoEM and qPCR measurement for Ion Torrent HBRR dataset.

Run	# reads	# mapped reads	*R* ^2^
LUC-140 265	1,588,375	1,142,306	0.728
POZ-124 266	1,495,151	1,066,809	0.729
DID-143 282	1,703,169	1,215,093	0.732
GOG-139 281	1,621,848	1,208,950	0.736
LUC-141 267	1,390,667	1,039,816	0.747

All runs	7,799,210	5,672,974	0.756

We also compared IsoEM and Cufflinks on two of the cancer Ion Torrent datasets, GOG-382 and DID-413. Methods were evaluated by calculating correlation between estimated FPKMs for External RNA Controls Consortium (ERCC) spike-in controls [38] with the known frequencies of these RNA controls in the samples (ERCC mix1 was spiked in for both runs). Table 7 presents the results of this comparison, showing higher *R*^2 ^for IsoEM in both cases.

**Table 7:** Correlation (*R*^2^) between known frequencies of spiked in ERCC controls and gene expression levels estimated by IsoEM and Cufflinks for Ion Torrent HeLa datasets.

Dataset	IsoEM	Cufflinks
GOG-482	0.723	0.683
DID-413	0.890	0.870

## Conclusion

In this paper we described the MaLTA-IsoEM method for simultaneous transcriptome assembly and quantification from Ion Torrent RNA-Seq data. Our approach explores transcriptome structure and incorporates a maximum likelihood model into the assembly and quantification procedure. Results on real cancer and MAQC RNA-Seq datasets show that Ion Torrent RNA-Seq data can be successfully used for transcriptome analysis. Transcriptome assembly and quantification accuracy was confirmed by comparison to annotated transcripts and TaqMan qRT-PCR measurements and External RNA Controls Consortium RNA spike-in controls. Experimental results on both real and synthetic datasets generated with various sequencing parameters and distribution assumptions suggest increased transcriptome assembly and quantification accuracy of MaLTA-IsoEM compared to existing state-of-the-art approaches.

## Competing interests

DB is a member of the Ion Bioinformatics group at Life Technologies Corporation. The work of S.M., A.C., S.A.S., I.M. and A.Z. was supported in part by Life Technology Grants "Novel transcript reconstruction from Ion Torrent sequencing" and "Viral Metagenome Reconstruction Software for Ion Torrent PGM Sequencer". The authors recognize the presence of potential conflicts of interest and affirm that the results reported in this paper represent original and unbiased observations.

## Authors' contributions

S.M., D.B., I.M. and A.Z. conceived the idea. S.M. designed algorithms, developed software, performed analysis and experiments, wrote the paper. A.C. performed analysis and experiments, wrote the paper. S.A.S. developed software, performed analysis and experiments, wrote the paper. D.B. contributed to designing the algorithms and writing the paper. I.M. contributed to designing the algorithms and writing the paper. A.Z. designed the algorithms, wrote the paper and supervised the project. All authors have read and approved the final manuscript.

## Declarations

Publication costs for this work were funded by the corresponding authors' institutions.

This article has been published as part of *BMC Genomics *Volume 15 Supplement 5, 2014: Selected articles from the Third IEEE International Conference on Computational Advances in Bio and Medical Sciences (ICCABS 2013): Genomics. The full contents of the supplement are available online at http://www.biomedcentral.com/bmcgenomics/supplements/15/S5.
